# Clinical Characterization of Autoimmune Hepatic Involvement in Sjogren’s Disease: A Retrospective Cohort Study in Korea

**DOI:** 10.3390/ijms26125734

**Published:** 2025-06-15

**Authors:** Youngjae Park, Jennifer Jooha Lee, Ji Hyeon Ju, Wan-Uk Kim, Sung-Hwan Park, Seung-Ki Kwok

**Affiliations:** Division of Rheumatology, Department of Internal Medicine, Seoul St. Mary’s Hospital, College of Medicine, The Catholic University of Korea, Seoul 06591, Republic of Korea; elwin84@catholic.ac.kr (Y.P.); poohish@catholic.ac.kr (J.J.L.); juji@catholic.ac.kr (J.H.J.); wan725@catholic.ac.kr (W.-U.K.); rapark@catholic.ac.kr (S.-H.P.)

**Keywords:** Sjogren’s disease, extraglandular manifestations, liver involvement, autoimmune hepatitis, primary biliary cholangitis

## Abstract

Sjogren’s disease (SjD) is a systemic autoimmune disease primarily affecting the exocrine glands. Systemic manifestations, including hepatic involvement, are increasingly recognized. This study aimed to delineate the clinical features and associated factors of autoimmune hepatic involvement in SjD. A retrospective analysis was conducted on patients diagnosed with SjD at Seoul St. Mary’s Hospital over the past 10 years. Autoimmune hepatic involvement was defined by fulfilling diagnostic criteria for autoimmune hepatitis (AIH) or primary biliary cholangitis (PBC). Clinical, serological, and demographic data were obtained from medical records. Among 1119 patients with SjD, 51 (4.6%) had autoimmune hepatic involvement. AIH (64.7%) was the most common type, followed by PBC (27.5%) and overlapping disease (7.8%). Compared to those without hepatic involvement, affected patients were older at diagnosis (*p* = 0.003) and showed higher frequencies of thrombocytopenia, splenomegaly, anti-centromere antibody (ACA), and elevated antinuclear antibody titers as measured by indirect immunofluorescence (IFI-HEp-2) (all *p* < 0.001). Multivariable analysis identified splenomegaly, elevated IFI-HEp-2 titer, and ACA positivity as independent factors associated with hepatic involvement. Most patients responded well to immunosuppressive therapy, with only a small proportion (15.7%) progressing to liver fibrosis. Autoimmune hepatic involvement is relatively uncommon but clinically meaningful in patients with SjD.

## 1. Introduction

Sjogren’s disease (SjD), previously known as Sjogren’s syndrome, is a chronic, systemic autoimmune disease predominantly affecting middle-aged women [1]. It is characterized by lymphocytic infiltration of the exocrine glands, leading to glandular dysfunction and classic sicca symptoms such as xerostomia and keratoconjunctivitis sicca [1,2]. However, over recent decades, SjD has come to be recognized not merely as a gland-limited disorder but as a systemic autoimmune condition with diverse extraglandular manifestations (EGMs) [3,4]. These include involvement of the lungs, kidneys, skin, peripheral and central nervous systems, and notably, the hepatobiliary system [3].

Among these systemic features, hepatic involvement in SjD has drawn increasing attention, particularly due to its frequent association with other autoimmune liver diseases. Abnormal liver function test results are not uncommon in patients with SjD, and a subset of these individuals go on to be diagnosed with autoimmune liver diseases such as primary biliary cholangitis (PBC) or autoimmune hepatitis (AIH) [5,6]. Early studies suggested hepatic abnormalities in SjD were infrequent or mild, often attributed to non-specific causes. However, more recent investigations have reported a broader spectrum of hepatic involvement, encompassing both asymptomatic biochemical abnormalities and overt autoimmune liver diseases with potential for progressive fibrosis and liver dysfunction [6,7,8].

The reported prevalence of hepatic abnormalities among SjD patients varies widely, from as low as 4% to over 40%, depending on the study design, definitions applied, and whether asymptomatic cases or imaging findings were included [6,7,8,9]. Moreover, hepatic involvement may precede or follow the diagnosis of SjD, further complicating clinical interpretation. Immunologically, these patients often present with distinct serologic profiles, including positivity for anti-mitochondrial antibody (AMA), anti-smooth muscle antibody (ASMA), anti-centromere antibody (ACA), and elevated antinuclear antibody (ANA) titers [6,9]. Certain systemic features, such as thrombocytopenia and splenomegaly, may also co-occur and provide clues to underlying liver involvement [6].

Importantly, hepatic involvement in SjD is more than just a laboratory abnormality. It may have prognostic implications and influence therapeutic decision-making. While most patients respond favorably to immunosuppressive treatment, a subset may progress to clinically significant liver disease, including fibrosis or cirrhosis, especially in the context of AIH or PBC. Despite this clinical relevance, hepatic manifestations in SjD remain under-recognized in routine rheumatologic care, and standardized screening strategies are lacking. This gap is particularly evident in Asian populations, including Korea, where large-scale studies examining hepatic involvement in SjD are limited.

Therefore, the present study was conducted to investigate the prevalence, clinical features, and associated immunological factors of autoimmune hepatic involvement in Korean patients with SjD. By analyzing a large cohort from a tertiary referral center, we aimed to provide a comprehensive characterization of this underexplored aspect of SjD, with the goal of improving clinical recognition and informing future diagnostic and therapeutic strategies.

## 2. Results

### 2.1. Patient Characteristics and Comparison by Hepatic Involvement

A total of 1119 patients diagnosed with SjD were included in the analysis. Among them, 51 patients (4.6%) were identified as having autoimmune hepatic involvement (Table 1). Patients with hepatic involvement were significantly older at the time of SjD diagnosis compared to those without (median 55 vs. 51 years, *p* = 0.003). No differences were observed in sex distribution or body mass index between the two groups. There were no significant differences in sicca symptoms, focus score, or EULAR Sjögren’s Syndrome Disease Activity Index (ESSDAI) total score [10]. However, hematologic abnormalities differed. Thrombocytopenia was significantly more frequent in patients with liver involvement (21.6% vs. 6.6%, *p* < 0.001), while rates of leukopenia and anemia were similar. Patients with hepatic involvement had significantly higher titers of ANA as measured by indirect immunofluorescence on HEp-2 cells (IFI-HEp-2) (*p* < 0.001), and a greater proportion had high IFI-HEp-2 titers (>1:640, 70.6% vs. 42.5%, *p* < 0.001). Additionally, ACA positivity was markedly more frequent in the liver involvement group (27.5% vs. 8.1%, *p* < 0.001). No significant differences were found in the prevalence of anti-Ro antibody, anti-La antibody, rheumatoid factor, complement levels, hypergammaglobulinemia, or cryoglobulin.

### 2.2. Extraglandular Manifestations

Among EGMs (Table S1), splenomegaly was significantly more frequent in the hepatic involvement group (Table 2, 3.9% vs. 0.1%, *p* < 0.001). Other systemic features such as arthritis, Raynaud’s phenomenon, lymphadenopathy, pulmonary involvement, peripheral neuropathy, and autoimmune thyroid disease did not differ significantly between groups. No patient in the liver involvement group was diagnosed with lymphoma.

### 2.3. Factors Associated with Hepatic Involvement: Univariable and Multivariable Analyses

In univariable logistic regression analysis (Table 3), age at diagnosis (odds ratio [OR] 1.03, 95% confidence interval [CI] 1.00–1.06, *p* = 0.003), thrombocytopenia (OR 3.92, 95% CI 2.00–8.08), IFI-HEp-2 titer (OR 1.00, 95% CI 1.00–1.00), high IFI-HEp-2 titer >1:640 (OR 3.25, 95% CI 1.74–5.84), ACA positivity (OR 4.39, 95% CI 2.30–8.37), and splenomegaly (OR 35.94, 95% CI 4.65–277.60) were significantly associated with liver involvement (all *p* < 0.001 except age at diagnosis). Multivariable logistic regression identified splenomegaly (adjusted OR 31.19, 95% CI 2.36–411.34, *p* = 0.008), ACA positivity (adjusted OR 2.58, 95% CI 1.22–5.42, *p* = 0.012), and IFI-HEp-2 titer (adjusted OR 1.00, 95% CI 1.00–1.00, *p* = 0.031) as independent predictors of hepatic involvement (Table 3).

### 2.4. Clinical Features by Subtype of Hepatic Involvement

Among the 51 patients with autoimmune hepatic involvement (Table 4), 33 were diagnosed with AIH (64.7%), 14 with PBC (27.5%), and 4 with overlap syndrome (7.8%). Median age at liver involvement diagnosis was similar across groups. In 31 cases (60.7%), hepatic disease was diagnosed prior to or simultaneously with SjD, while in 20 cases (39.2%), it was diagnosed after SjD. The median interval from SjD diagnosis to hepatic involvement was shorter in PBC patients (2 months) than in those with AIH (8 months). Liver enzyme abnormalities were more frequent in the AIH group, with 66.7% showing alanine aminotransferase (ALT) elevation and 63.6% showing gamma-glutamyl transpeptidase (gGTP) elevation. PBC patients showed lower rates of ALT, alkaline phosphatase (ALP) and gGTP elevation. Although the dominant IFI-HEp-2 pattern was generally similar across different types of liver involvement, the speckled pattern was slightly more frequent in patients with AIH (51.5%). AMA positivity was found in 71.4% of PBC patients, whereas ASMA and anti-Liver-Kidney Microsomal type 1 (LKM1) antibody were mostly negative. Notably, ACA was detected in 42.9% of PBC patients, and in 20.7% of AIH patients. Hepatoprotective agents, such as ursodeoxycholic acid (UDCA), silymarin, and a carnitine-based metabolic compound, were commonly used across all subtypes. Immunosuppressive therapy was more frequently used in AIH patients (39.4%) than in PBC patients (7.1%). Among patients with follow-up data, most showed favorable responses, and only a minority (15.7%) developed liver fibrosis.

## 3. Discussion

In this large retrospective cohort of 1119 patients with SjD, autoimmune hepatic involvement was identified in 4.6% of cases, with AIH being more common than PBC. Patients with hepatic involvement were more likely to present with thrombocytopenia, splenomegaly, high IFI-HEp-2 titer, and ACA positivity. Multivariable analysis confirmed splenomegaly, high IFI-HEp-2 titer, and ACA as independent predictors of liver involvement.

In this study, autoimmune hepatic involvement was identified in 4.6% of the cohort (Table 1). This prevalence is notably lower than that reported in previous studies, which have ranged from approximately 6% to over 20%, particularly in cohorts enriched for patients with abnormal liver function tests or preexisting suspicion of hepatic disease [7,8,9]. Unlike those studies, our cohort comprised an unselected population of SjD patients, more closely representing the general clinical spectrum seen in real-world rheumatology practice. The relatively low prevalence observed here may thus reflect a more accurate estimate of clinically significant hepatic involvement in SjD. Interestingly, AIH was more frequently observed than PBC in our cohort, contrasting with the majority of prior studies in which PBC has been reported as the predominant autoimmune liver disease in association with SjD [7,8]. Several explanations may account for this divergence. First, ethnic and geographic differences in the distribution of autoimmune liver diseases may influence disease expression. A previous study in Korean populations have reported higher rates of AIH than PBC, consistent with our findings [11]. Second, the diagnostic threshold for AIH, especially with simplified criteria emphasizing serologic markers, may facilitate earlier or more frequent detection, particularly in the absence of liver biopsy [12]. On the other hand, PBC, particularly AMA-negative or early-stage forms, may be under-recognized due to less prominent symptoms or biochemical findings. The identification of overlap syndromes in our study further supports the concept that autoimmune hepatic involvement in SjD spans a spectrum, rather than fitting neatly into traditional diagnostic categories.

Our analysis revealed several clinical and serological features significantly associated with hepatic involvement in SjD. Patients with liver involvement were older at the time of SjD diagnosis and exhibited significantly higher frequencies of thrombocytopenia, high IFI-HEp-2 titer, ACA positivity, and splenomegaly (Table 1 and Table 2). Among these, splenomegaly and ACA positivity remained independently associated in multivariable analysis (Table 3). Although age at diagnosis and thrombocytopenia showed significant associations in the univariable analysis, they did not remain significant in the multivariable model. This may be explained by overlapping or related clinical features, as thrombocytopenia often coexists with splenomegaly and older age may be indirectly associated with serologic markers such as ACA positivity. These factors may act as stronger predictors when considered together, diminishing the independent effects of age and thrombocytopenia in the adjusted model. Splenomegaly, although rare in the overall cohort, was strikingly more frequent in patients with hepatic involvement, suggesting potential underlying portal hypertension or immune-mediated splenic activation. While most previous studies have not systematically examined splenomegaly in SjD, our findings imply that this clinical finding may serve as an important clue prompting further hepatic evaluation. ACA, traditionally associated with PBC and limited systemic sclerosis, was also significantly linked to hepatic involvement in our SjD patients. This is consistent with previous observations of ACA in AMA-negative PBC and other atypical autoimmune liver disease phenotypes [13,14,15]. Notably, ACA was not confined to PBC cases but was also observed in a subset of AIH patients, suggesting that its presence may reflect a broader autoimmune predisposition within the hepatobiliary axis [16]. Given that ACA is classically linked to limited systemic sclerosis, we explored whether Raynaud’s phenomenon, a hallmark feature of that condition, was more frequent among patients with hepatic involvement or ACA positivity. However, as shown in Table 2, the frequency of Raynaud’s phenomenon did not differ significantly between patients with and without hepatic involvement. The routine testing for ACA in SjD patients is not yet standardized, but our findings highlight its potential role as a serologic marker that warrants closer hepatic monitoring [17].

The clinical profiles of hepatic involvement differed by subtype. Patients with AIH tended to have greater elevations in transaminases such as ALT and gGTP, consistent with hepatocellular injury, and were more likely to receive immunosuppressive therapy (Table 4). This difference in treatment patterns reflects the established therapeutic strategies for these two conditions. AIH typically requires immunosuppressive therapy, including corticosteroids and/or azathioprine, as the mainstay of management. In contrast, PBC is primarily treated with UDCA, and immunosuppressive agents are not routinely used unless there is overlap with AIH or poor response to standard therapy. Given the clinical relevance of hepatic involvement in SjD, therapeutic decisions should be guided by the underlying liver disease subtype. In AIH, prompt initiation of immunosuppressive therapy, usually with prednisone alone or in combination with azathioprine, is strongly recommended to prevent disease progression [12]. On the other hand, UDCA remains the first-line treatment for PBC, and the role of immunosuppressants is limited to specific scenarios, such as overlap syndromes or UDCA nonresponse [18,19]. In clinical practice, a multidisciplinary approach involving both rheumatologists and hepatologists is essential for accurate diagnosis, biopsy consideration, and individualized treatment planning. Early recognition and appropriate intervention may improve liver-related outcomes in SjD patients with hepatic involvement. Treatment response was generally favorable across all subtypes, with most patients responding to supportive care or immunosuppression and only a minority progressing to liver fibrosis (Table 4). These findings are encouraging and underscore the importance of timely recognition and management to prevent long-term hepatic sequelae.

Our study has several limitations. The retrospective design inherently introduces potential for bias due to incomplete documentation and heterogeneity in testing. Some cases of subclinical or early-stage hepatic involvement may have been missed, especially in patients without comprehensive liver imaging or biopsy. Second, the study population was derived from a single tertiary center in Korea, which may limit generalizability to other geographic or ethnic populations. Third, although established criteria were used to define hepatic involvement, not all patients had histologic confirmation, particularly those with AMA-negative profiles or borderline findings. Finally, we did not assess long-term hepatic outcomes beyond fibrosis, such as progression to cirrhosis or hepatocellular carcinoma.

In conclusion, autoimmune hepatic involvement is an uncommon but clinically significant manifestation in patients with SjD. Our findings demonstrate that AIH may be more prevalent than PBC in East Asian SjD populations, and that certain clinical and serologic markers, particularly splenomegaly and ACA positivity, are useful predictors of hepatic involvement. These features may help guide clinicians in selecting SjD patients who would benefit from further hepatic evaluation. Future prospective, multicenter studies with standardized hepatic assessment protocols, including liver histopathology and long-term follow-up, are needed to validate these findings and refine screening strategies for autoimmune liver disease in SjD.

## 4. Materials and Methods

### 4.1. Study Design and Population

This was a retrospective cohort study performed at Seoul St. Mary’s Hospital, a tertiary referral center in South Korea. Electronic medical records of patients diagnosed with SjD between January 2013 and December 2022 were reviewed. The classification of SjD was based on the 2016 American College of Rheumatology/European League Against Rheumatism classification criteria [20]. Patients with SjD overlapped with systemic lupus erythematosus, rheumatoid arthritis, systemic sclerosis, or other defined autoimmune connective tissue diseases were excluded.

### 4.2. Definition of Autoimmune Hepatic Involvement

Autoimmune hepatic involvement was defined as the presence of AIH or PBC, based on the fulfillment of established international diagnostic criteria. AIH was diagnosed according to the simplified criteria proposed by the International Autoimmune Hepatitis Group [12], incorporating elevated serum transaminase levels, hypergammaglobulinemia, autoantibody positivity (ANA, ASMA, or LKM-1), and compatible histopathology when available. Liver biopsy was performed selectively, based on clinical judgment and hepatology consultation, particularly in cases where the diagnosis of AIH was uncertain or atypical features were present. PBC was defined based on persistent cholestatic liver enzyme elevation, presence of AMA, and/or compatible histologic findings [18,19]. Patients with features of both AIH and PBC were categorized as having AIH-PBC overlap syndrome. The timing of hepatic involvement diagnosis was recorded in reference to the date of SjD diagnosis.

### 4.3. Data Collection

Demographic data included age at SjD diagnosis, sex, and body mass index. Clinical features included glandular symptoms, focus score from minor salivary gland biopsy, and systemic disease activity using the ESSDAI [10,21]. EGMs including arthritis, Raynaud’s phenomenon, lymphadenopathy, splenomegaly, pulmonary involvement, cutaneous vasculitis, myositis, peripheral neuropathy, CNS involvement, thyroid disease, and kidney involvement were recorded based on the fulfillment of pre-defined criteria (Table S1). Hematological abnormalities included leukopenia (WBC < 4000/μL), anemia (Hb < 11.0 g/dL), and thrombocytopenia (platelets < 150,000/μL). Laboratory parameters included levels of C3, C4, and total gamma globulin. Immunologic markers evaluated were IFI-HEp-2 titer (with high positivity defined as >1:640), anti-Ro antibody (defined as positivity for either anti-Ro52 or anti-Ro60), anti-La antibody, ACA, and rheumatoid factor. Hypergammaglobulinemia was defined as serum gamma globulin >1.6 g/dL.

### 4.4. Statistical Analysis

All statistical analyses were conducted using IBM-SPSS Statistics version 24.0 (SPSS Inc., Chicago, IL, USA). Continuous variables were expressed as a median (interquartile range) and categorical variables as a number (percentage). Intergroup comparisons between patients with and without hepatic involvement were made using the Mann–Whitney U test for continuous variables and the chi-square test or Fisher’s exact test for categorical variables. Univariable logistic regression analysis was first performed to assess factors associated with hepatic involvement. Variables with *p* < 0.05 in univariable analysis were subsequently included in a multivariable logistic regression model. ORs and 95% CIs were calculated to assess the strength of associations. A two-sided *p* value < 0.05 was considered statistically significant.

## Figures and Tables

**Table 1:** Demographic and clinical information of patients with Sjogren’s disease.

	Overall, *n* = 1119	With Hepatic Involvement, *n* = 51	Without Hepatic Involvement, *n* = 1068	*p* Value
Age at diagnosis, years	51 (41–59)	55 (50–62)	51 (41–58)	0.003
Sex, female	1097 (98.0)	50 (98.0)	1047 (98.0)	>0.999
Body mass index, kg/m^2^	21.7 (19.8–23.2)	21.8 (20.8–23.5)	21.8 (20.1–23.4)	0.342
Dry eyes	971 (86.8)	41 (80.4)	935 (87.5)	0.200
Dry mouth	995 (88.9)	46 (90.2)	953 (89.2)	0.988
Focus score	2 (1–4)	2 (2–2)	2 (2–2)	0.836
ESSDAI total score	2 (1–5)	2 (1–5)	3 (1–6)	0.692
Leukopenia	288 (25.7)	11 (21.6)	277 (25.9)	0.594
Anemia	208 (18.6)	10 (19.6)	198 (18.5)	0.994
Thrombocytopenia	81 (7.2)	11 (21.6)	70 (6.6)	<0.001
IFI-HEp-2 positivity	1041 (93.0)	51 (100)	990 (92.7)	0.085
IFI-HEp-2 titer	400 (200–1280)	800 (400–2560)	400 (200–800)	<0.001
High IFI-HEp-2 titer (>1:640)	490 (43.8)	36 (70.6)	454 (42.5)	<0.001
Anti-Ro antibody positivity	882 (78.8)	38 (74.5)	851 (79.7)	0.474
Anti-La antibody positivity	359 (32.1)	14 (27.5)	345 (32.3)	0.568
Anti-centromere antibody positivity	100 (8.9)	14 (27.5)	86 (8.1)	<0.001
Rheumatoid factor positivity	612 (54.7)	26 (51.0)	586 (54.9)	0.688
Low C3	157 (14.0)	3 (5.9)	154 (14.4)	0.383
Low C4	46 (4.1)	2 (3.9)	44 (4.1)	>0.999
Hypergammaglobulinemia	400 (35.7)	22 (43.1%)	378 (35.4)	0.328
Cryoglobulin	5 (0.4)	0 (0.0)	5 (0.5)	>0.999

Data are shown as *n* (%) or median (interquartile range). ESSDAI: European League Against Rheumatism Sjogren’s syndrome disease activity index, IFI-HEp-2: indirect immunofluorescence on HEp-2 cells.

**Table 2:** Extraglandular manifestations of patients with Sjogren’s disease.

	Overall, *n* = 1119	With Hepatic Involvement, *n* = 51	Without Hepatic Involvement, *n* = 1068	*p* Value
Arthralgia/arthritis	472 (42.2)	17 (33.3)	455 (42.6)	0.244
Raynaud’s phenomenon	187 (16.7)	9 (17.6)	178 (16.7)	>0.999
Lymphadenopathy	108 (9.7)	4 (7.8)	104 (9.7)	0.838
Lymphoma	9 (0.8)	0 (0.0)	9 (0.8)	>0.999
Splenomegaly	3 (0.3)	2 (3.9)	1 (0.1)	<0.001
Pulmonary involvement	76 (6.8)	2 (3.9)	74 (6.9)	0.583
Cutaneous vasculitis	102 (9.1)	3 (5.9)	99 (9.3)	0.567
Myositis	2 (0.2)	1 (2.0)	1 (0.1)	0.165
Peripheral neuropathy	46 (4.1)	2 (3.9)	44 (4.1)	>0.999
Central nervous system disease	9 (0.8)	1 (2.0)	8 (0.7)	0.885
Autoimmune thyroid disease	128 (11.4)	10 (19.6)	118 (11.0)	0.099
Kidney involvement	21 (1.9)	1 (2.0)	20 (1.9)	>0.999

Data are shown as *n* (%).

**Table 3:** Univariable and multivariable analyses of factors associated with hepatic involvement in Sjogren’s disease.

	Univariable OR (95% CI)	*p* Value	Multivariable OR * (95% CI)	*p* Value
Age at diagnosis	1.03 (1.00–1.06)	0.003	1.06 (0.98–1.15)	0.144
Thrombocytopenia	3.92 (200–8.08)	<0.001	2.27 (0.80–6.42)	0.122
IFI-HEp-2 titer	1.00 (1.00–1.00)	<0.001	1.00 (1.00–1.00)	0.031
High IFI-HEp-2 titer	3.25 (1.74–5.84)	<0.001	1.37 (0.55–3.42)	0.504
Anti-centromere antibody positivity	4.39 (2.30–8.37)	<0.001	2.58 (1.22–5.42)	0.012
Splenomegaly	35.94 (4.65–277.60)	<0.001	31.19 (2.36–411.34)	0.008

* Variables with *p* < 0.05 in univariable analysis and clinically relevant covariates (sex and body mass index) were included in the multivariable logistic regression model. OR: odds ratio, CI: confidence interval.

**Table 4:** Clinical features of Sjogren’s disease patients with liver involvement.

	PBC, *n* = 14	AIH, *n* = 33	Combined, *n* = 4
Age at diagnosis of liver involvement, years	56 (51–64)	60 (51–64)	55 (46–58)
Diagnosis of liver involvement			
Simultaneously	3 (21.4)	3 (9.1)	0 (0.0)
Prior to SjD	5 (35.7)	16 (48.5)	4 (100.0)
After SjD	6 (42.9)	14 (42.4)	0 (0.0)
Time to liver involvement after SjD diagnosis, months	2 (0–10)	8 (1–50)	
ALT elevation	5 (35.7)	22 (66.7)	2 (50.0)
ALP elevation	5 (35.7)	15 (45.5)	2 (50.0)
gGTP elevation	7 (50.0)	21 (63.6)	4 (100.0)
Dominant IFI-HEp-2 pattern			
Speckled	5 (35.7)	17 (51.5)	1 (25.0)
Cytoplasmic	4 (28.6)	5 (15.2)	1 (25.0)
Centromere	5 (35.7)	8 (24.2)	2 (50.0)
Homogeneous	0 (0.0)	3 (9.1)	0 (0.0)
Anti-mitochondrial antibody positivity	10 (71.4)	5/32 (15.6)	4 (100.0)
Anti-LKM1 antibody positivity	0/8 (0.0)	0/19 (0.0)	0/3 (0.0)
Anti-smooth muscle antibody positivity	0/9 (0.0)	4/26 (15.4)	0 (0.0)
ANCA positivity	1/11 (9.1)	6/28 (21.4)	0 (0.0)
Anti-centromere antibody positivity	6 (42.9)	6/29 (20.7)	2/3 (66.7)
Treatment			
Steroid and/or immunosuppressants	1 (7.1)	13 (39.4)	0 (0.0)
Hepatoprotective agents *	12 (85.7)	31 (93.9)	4 (100.0)
Response, resolution	1/1 (100.0)	12/13 (92.3)	
Fibrosis	3 (23.1)	5 (15.2)	0 (0.0)

Data are shown as *n* (%) or median (interquartile range). * Hepatoprotective agents include ursodeoxycholic acid, silymarin, and a carnitine-based metabolic compound. PBC: primary biliary cholangitis, AIH: autoimmune hepatitis, SjD: Sjogren’s disease, ALT: alanine aminotransferase, ALP: alkaline phosphatase, gGTP: gamma-glutamyl transpeptidase, IFI-HEp-2: indirect immunofluorescence on HEp-2 cells, LKM1: Liver-Kidney Microsomal type 1, ANCA: anti-neutrophil cytoplasmic antibody.

## Data Availability

The raw data supporting the conclusions of this article will be made available by the authors on request.

## Supplementary Materials

The following supporting information can be downloaded at: https://www.mdpi.com/article/10.3390/ijms26125734/s1.

## Abbreviations

The following abbreviations are used in this manuscript:

SjD	Sjogren’s disease
EGMs	Extraglandular manifestations
PBC	Primary biliary cholangitis
AIH	Autoimmune hepatitis
AMA	Anti-mitochondrial antibody
ASMA	Anti-smooth muscle antibody
ACA	Anti-centromere antibody
ANA	Antinuclear antibody
ESSDAI	EULAR Sjögren’s Syndrome Disease Activity Index
IFI-HEp-2	Indirect immunofluorescence on HEp-2 cells
OR	Odds ratio
CI	Confidence interval
ALT	Alanine aminotransferase
gGTP	Gamma-glutamyl transpeptidase
ALP	Alkaline phosphatase
LKM1	Liver-Kidney Microsomal type 1
UDCA	Ursodeoxycholic acid
