# Supplementary material for: Clinical Characterization of Autoimmune Hepatic Involvement in Sjogren’s Disease: A Retrospective Cohort Study in Korea
Source: Int J Mol Sci. 2025 Jun 15;26(12):5734. doi: 10.3390/ijms26125734 (PMC12192730; doi:10.3390/ijms26125734)
Supplement: Supplementary file 1 [file ijms-26-05734-s001.zip › ijms-3679226-supplementary.pdf]

Supplementary Materials

Table S1. Definitions of each type of extraglandular manifestations.

**Items**	**Definitions**
Articular involvement	A history or presence of morning stiffness over 30 minutes or any swollen or tender joints with the exclusion of degenerative arthritis
Raynaud's phenomenon	The presence of at least two color changes in distal phalanges including pallor, cyanosis, and redness combined with sensory changes, such as pain and tingling
Lymphadenopathy	The presence of palpable lymph nodes over 1 cm
Lymphoma	A history of lymphoma confirmed by pathology
Splenomegaly	A palpable spleen below the left costal margin during full inspiration, or a splenic length ≥12 cm on imaging studies such as CT or ultrasound
Pulmonary involvement	A history or presence of autoimmune-mediated or interstitial lung disease
Cutaneous vasculitis	The presence of palpable purpura suggesting the extravasation of red blood cells into the dermis
Myositis	The presence of myositis diagnosed by elevated serum muscle enzymes, such as creatinine kinase and abnormal findings suggesting myositis in electromyography or biopsy
Peripheral neuropathy	Abnormal findings suggesting peripheral neuropathy in electroneurography
Central nervous system involvement	The presence of cranial or optic neuritis or cerebrovascular accident or epilepsy

Autoimmune thyroid disease	A history or presence of Graves’ disease or Hashimoto’s thyroiditis
Kidney involvement	The presence of interstitial nephritis combined with renal tubular acidosis or glomerulonephritis combined with proteinuria over 0.5 g/day
